# Retraction: PDIA6 promotes the proliferation of HeLa cells through activating the Wnt/β-catenin signaling pathway

**DOI:** 10.18632/oncotarget.28522

**Published:** 2024-03-14

**Authors:** Huijun Gao, Bing Sun, Hailu Fu, Xinming Chi, Faming Wang, Xiaoyu Qi, Jun Hu, Shujuan Shao

**Affiliations:** ^1^Department of Histology and Embryology, Dalian Medical University, Dalian, China; ^2^Department of Thoracic Surgery, The First Hospital of Dalian Medical University, Dalian, China; ^3^Key Laboratory of Proteomics, Dalian Medical University, Dalian, China; ^*^These authors have contributed equally to this work

**This article has been retracted:** Two instances of internal image duplication were discovered in this paper. Specifically, the β-actin blots in Figure 4A are duplicates of the β-actin blots in Figure 3A. The PDIA6 panel of Figure 5A is also a duplicate of the PDIA6 panel in Figure 2G. The authors were unable to make corrections using the original data. Therefore, in accordance with the decision of the Science and Technology Department of Dalian Medical University, and with the agreement of all authors, Oncotarget has decided to retract this paper.

Original article: Oncotarget. 2016; 7:53289–53298. 53289-53298. https://doi.org/10.18632/oncotarget.10795

